# Music Use for Sedation in Critically ill Children (MUSiCC trial): study protocol for a pilot randomized controlled trial

**DOI:** 10.1186/s40814-020-0563-x

**Published:** 2020-02-25

**Authors:** Gonzalo Garcia Guerra, Ari Joffe, Cathy Sheppard, Krista Hewson, Irina A. Dinu, Allan de Caen, Hsing Jou, Lisa Hartling, Sunita Vohra, the Canadian Critical Care Trials Group

**Affiliations:** 1 Department of Pediatrics, University of Alberta, College Plaza 8215 112 St NW, Edmonton, AB T6G 1C9 Canada; 2 Stollery Children’s Hospital, Edmonton, AB Canada; 3 Department of Educational Psychology, University of Alberta, Edmonton, AB Canada; 4 School of Public Health, University of Alberta, Edmonton, AB Canada

**Keywords:** Sedation, Analgesia, Intensive care, Pediatric, Music

## Abstract

**Background:**

Stress induced by pain and anxiety is common in pediatric intensive care unit (PICU) patients. Sedation/analgesia in PICU is usually achieved through various analgesics and sedatives. Excessive use of these drugs can put patients at risk for hemodynamic/respiratory instability, prolonged ventilation, withdrawal, delirium, and critical illness polyneuromyopathy.

The use of non-pharmacologic interventions has been recommended by sedation guidelines. However, non-pharmacological measures in PICU, including music and noise reduction, have been inadequately studied.

**Methods:**

The Music Use for Sedation in Critically ill Children (MUSiCC trial) pilot study is an investigator-initiated, three-arm, randomized controlled trial (RCT) on the use of music for sedation in PICU. The main goal of the study is to demonstrate feasibility of a music trial in PICU and to obtain the necessary information to plan a larger trial. The study compares music versus noise cancelation versus control in sedated and mechanically ventilated children admitted to PICU. In the music group, children receive the music (modified classical music) three times a day for 30 min at a time. Music is delivered with noise cancelation headphones. The noise cancelation group receives the same intervention but with a no music (sham playlist). The control group receives usual care with no specific intervention. Children remain in the study until extubation or a maximum of 7 days. The primary outcomes of the study are feasibility and sedation/analgesia requirements. Secondary outcomes include change in vital signs before and during the intervention, ICU delirium, and adverse effects related to the intervention. The estimated sample size is 20 subjects per group for a total of 60 children.

**Discussion:**

Despite being recommended by current guidelines, evidence to support the use of music in PICU is lacking. Music has the potential to reduce sedation requirements and their negative side effects. This pilot RCT will demonstrate feasibility and provide the necessary information to plan a larger trial focusing on the effectiveness of the intervention.

**Trial registration:**

The study was registered at ClinicalTrials.gov (NCT03497559) on April 13, 2018.

## Background

Stress induced by pain and anxiety is common in pediatric intensive care unit (PICU) patients and can impede the care to children as well as their recovery [1]. Children in PICUs experience pain and anxiety for a wide variety of reasons [2]. In PICU, sedation/analgesia is important not just for comfort, but also for safety. Children with inadequate sedation/analgesia are at risk for loss of vascular access, extubation, self-injury, post-traumatic stress, etc. Sedation/analgesia in PICU is usually achieved through various analgesics and sedatives. However, excessive use of these drugs can put patients at risk for hemodynamic/respiratory instability, prolonged ventilation, withdrawal, delirium, and critical illness polyneuromyopathy. These negative consequences can lead to prolonged PICU stay and increase healthcare costs [2–4].

Non-pharmacologic measures for analgesia/anxiolysis are interventions that do not involve drugs, and thus may reduce the total medication requirement and their side effects [4]. The use of non-pharmacologic interventions has been recommended by published international sedation guidelines [5, 6]. However, none of these guidelines state how these interventions should be provided. A survey conducted by our group showed that Canadian PICUs do not use them routinely [7]. Non-pharmacological measures in PICU, including music and noise reduction, have been inadequately studied [8]. Even more, the need for research around non-pharmacological interventions in PICU has been recently identified [7]. In our survey, 85% of the respondents stated that non-pharmacological interventions in PICU should be formally studied.

### Music and medicine, mechanism of action

Although music has been used for years in healthcare, the exact mechanisms by which it can reduce pain/anxiety are not well understood. It is known that music can modify emotional state by releasing anti-stress hormones and by activating the limbic system of the brain [9]. According to the gate control theory of pain, distracters such as music can block certain neural pathways and diminish the amount of perceived pain [9–13].

### Music in adult intensive care units

A systematic review on the use of music in mechanically ventilated adults found that music was associated with lower levels of anxiety, lower sedation requirements, and improved vital signs suggesting relaxation [14]. A randomized controlled trial (RCT) on patient-directed music demonstrated that music was associated with a reduction in anxiety and in sedation requirements in critically ill adults [13]. A recent study showed that music can also improve sleep in adult ICU patients [15].

### Music in the pediatric setting

A systematic review demonstrated that music can reduce procedural pain in a variety of clinical settings [10]. Another review showed that music was associated with lower pain scores and anxiety in children going for surgery [8]. Other studies have found similar results showing that music can be used to treat pain in pediatric clinical settings [12, 16, 17]. However, none of these studies explored the use of music for sedation/analgesia in the intensive care unit setting other than during a single painful procedure.

### Music in pediatric intensive care

In newborns, music has been shown to be effective in reducing pain and stress behavior during procedures. Music is also associated with more stable vital signs, increased weight gain, shorter length of stay, and increased parental satisfaction with neonatal intensive care [17–21]. A large RCT confirmed that music is associated with better vital signs, improved feeding behavior, and prolonged time remaining settled [22]. Except for studies conducted in neonatal intensive care units, there is only one RCT on music in critically ill children that evaluated the effects of music on vital signs and pain scores, which demonstrated that music improved these clinical signs [16]. However, this trial used music only once for 30 min in the first 24 h after surgery and did not investigate effects on sedation requirements. Whether these benefits would be observed with repeated use over several days in PICU is not known.

### Potential concerns in critically ill children

Although music can have positive effects, the contrary is also possible [23]. There is evidence that pleasant music can alleviate pain perception, but unpleasant music had no significant effect [24, 25]. Music, especially with the use of headphones, can pose a challenge for patient communication [12]. As communication is already limited in PICU patients, close monitoring while applying this type of interventions may be required. As recommended by the American Academy of Pediatrics, volume should be kept < 45 dB [26]. Music may reduce pain and anxiety, but patients should not receive music as the sole source of sedation as it is not likely to be adequate in isolation [27].

## Rationale for the study and study hypothesis

Despite the recommendations from current guidelines on the use of music in critical care, a recent systematic review conducted by the authors [28] demonstrated that there are no published or ongoing RCTs investigating the effect of music on sedation and analgesia requirements in critically ill children [5, 6]. Hence, the effect of music in sedated and mechanically ventilated children and the optimal administration of such an intervention is unknown. Previous studies, especially in adult ICU, have led the way on the use of music to provide sedation/analgesia in the critical care setting. However, the optimal administration of music (type, mode, and frequency) and its effectiveness in PICU needs to be established. The aim of the MUSiCC pilot trial is to determine the feasibility of a pediatric music trial, to study the effects of music on sedation/analgesia requirements and in the incidence of delirium in children admitted to PICU. We hypothesize that an RCT of music in critically ill children will be feasible. Further, the pilot study will allow us to collect pediatric data on sedation and analgesia requirements, which will be necessary to calculate the sample size for a future, larger, trial. A survey conducted by this research group found that reduction in sedation requirements is a meaningful and clinically relevant outcome for a trial on non-pharmacologic interventions in PICU [7]. The study is currently being conducted in the PICU and Pediatric Cardiac Intensive Care Unit (PCICU) of the Stollery Children’s Hospital (Edmonton, AB, Canada).

## Methods

### Study design

The MUSiCC trial pilot study is an investigator-initiated, three-arm RCT examining the use of music for sedation in PICU. A parallel three-group design including a noise cancelation group was included based on adult data showing that noise cancelation can reduce sedation requirements as well as pediatric evidence that noise levels are associated with sedation requirements in PCICU [13, 29].

### Patient eligibility—inclusion criteria

Upon admission to the PICU or PCICU, all critically ill children are screened for eligibility and inclusion in the MUSiCC pilot trial by research nurses. All non-eligible patients, identified by the investigators, are logged. All children admitted to the Stollery Children’s Hospital PICU or PCICU, with an age of 1 month to 16 years of age, receiving invasive mechanical ventilation for > 24 h, are eligible and approach for consent by our research nurses.

### Exclusion criteria

Patients meeting one or more of the following criteria are excluded:

- Known hearing deficit
- Infants < 1 month old and/or < 3 kg (as the headphones will not fit)
- Major cranial-facial abnormalities (as the headphones will not fit)
- Traumatic brain injury (could cause pain in cranial fractures and risk of displacing intracranial catheters)
- Not receiving any sedation and/or analgesia drugs
- Receiving paralytic agents
- Expected to die in the next 48 h
- On extracorporeal membrane oxygenation (ECMO) with neck cannulation (difficulty fitting the headphones and risk of cannula displacement)
- Enrolled in another sedation intervention study

### Data collection at study entry

At baseline, the following variables are being recorded: demographic variables (sex, weight, age, diagnosis), unit of admission, operative status, pediatric risk of mortality (PRISM) score and whether the patient was on sedation and/or analgesia drugs prior to ICU admission. At the time of enrolment, we are also collecting information on the following variables: pediatric logistic organ dysfunction-2 (PELOD-2) score, inotrope score, need for invasive procedures, presence of invasive lines and tubes [30]. Variables are recorded in an anonymized database using REDCap, Research Electronic Data Capture [31]. The data collection case report form is attached as Additional file 1.

## Randomized treatment allocation

### Randomization procedure and treatment allocation

Randomization is done by a computer-based program to ensure allocation concealment and is being performed by the Epidemiology Coordinating and Research Centre (EPICORE), a clinical trial unit at the University of Alberta. A total of 60 patients are being consecutively randomly assigned in a 1:1:1 ratio to receive music, noise cancelation, or control.

### Blinding

In order to blind the intervention, the research nurse provides the portable music player (Apple iPod^TM^ touch, California, USA) with music or silent recording based on group allocation and does not disclose this information to the healthcare team or the family. The iPods assigned to the noise cancelation group have a sham playlist with a silent recording that displays in the iPod screen as if music were being played. Each 30 min playlist (music and sham) starts with 1 min of silent recording in an attempt to maintain blinding of the intervention. The volume in the iPods is set at approximately 45 to 55 dB. Based on the nature of the intervention, it is impossible to blind the use of headphones vs. control. However, collection of outcome data is blinded to group allocation. The statistician analyzing the data will also be blinded to the group allocation.

### Randomized interventions

After consent and randomization, patients are started on the assigned intervention (music/noise cancelation/control) 24-48 h after admission to the PICU. In the music and noise cancelation groups, the intervention is delivered three times a day for 30 min at a time. The bedside nurse determines the exact time of each intervention so that it does not interfere with care, e.g., avoiding times when clinical interventions are taking place. However, the bedside nurse is asked to deliver each intervention within the following time windows: 7 am-12 pm (morning intervention), 12-4 pm (afternoon intervention), and 4 pm-8 pm (evening intervention). The control group receives usual care. Music is delivered with the use of noise cancelation headphones (PURO® Sound Labs Kids BT2200 and BT5200, California, USA) and an iPod touch. Puro Sound Labs headphones have an intrinsic volume restrictor of 85 dB and 82% ambient noise cancelation and have two different sizes that allow to deliver the intervention across a wide range of ages. Music selection was performed by our music therapist (KH) and consists of short pieces of classical music with a tempo of around 60 beats per minute with preference for major keys and with attention to avoid dramatic moments, unsettling chords, and minor keys, as they can be associated with sadness. We created four different music playlists of 30 min each to add variation to the intervention. In the noise cancelation group, the intervention is provided with the same headphones connected to an iPod with a sham playlist with silent recording as described above. Children are assessed with the Sedation Behavior Scale (SBS) before and during the intervention [32]. Signs of agitation or an increase in the SBS by two points indicate failure of the intervention. Patients are to remain on protocol as long as they are receiving invasive mechanical ventilation or for a maximum of 7 days, whichever comes first.

### Concomitant interventions

Other than the music/noise cancelation interventions, clinical care is not protocolized and is according to the usual management. Sedation and analgesia management is not directed by the study protocol; it is up to the attending PICU physician to decide the drugs, dose, and intervals to provide comfort and analgesia to enrolled patients. Assessment of the patients’ sedation status and withdrawal symptoms is conducted every 6 h by the bedside nurse as part of the routine care. Sedation status is assessed with the use of the SBS and withdrawal is assessed with the Withdrawal Assessment Tool (WAT-1) score; both are well validated tools [32–34].

## Handling of re-admissions to the PICU

Patients re-admitted to the PICU are considered eligible for enrolment as long as they required invasive mechanical ventilation and the use of sedation and/or analgesia upon their re-admission. Patients and families are re-approached for consent and randomization and started on the new assigned intervention (music/noise cancelation/control) within 24-48 h of their new admission.

## Outcome measures

### Primary endpoints

The primary outcomes of this trial are feasibility and sedation requirements. In order to determine feasibility of a music trial in critically ill children, we are collecting information on: number of eligible patients, number of patients enrolled, rate of enrolment, time to complete participation, protocol adherence, and reasons for protocol deviation. Feasibility is defined as a protocol adherence of 80% and consent rate of 70%, with an average enrolment rate of 5 patients per month. Protocol adherence is defined as receiving the allocated intervention for 30 min three times per day during the time patient remains in the study.

Information on sedation and analgesia requirements will allow the appropriate sample size calculations for a larger trial if this study demonstrates that a music intervention in critically ill children is feasible. A survey conducted by this research group found that reduction in sedation requirements is a meaningful and clinically relevant outcome for a trial on non-pharmacologic interventions in PICU [7]. Sedation requirements will be captured as a daily intensity score and intermittent dose (PRN) frequency [13]. The sedative drug intensity score aggregates the amount of sedation/analgesia from different drug classes using a weight-adjusted dose of each sedative administered during 4-h time blocks [13]. Every sedation amount for each drug is then placed in quartiles created by using the patients’ data during the time the patients are involved in the study. The values are then summed over the six 4-h blocks to obtain the daily score. Dose frequency will be captured by the administration of a (PRN) dose of any of the sedative drugs. This way of capturing sedation requirements allows to account for the administration of different and non-equivalent types of drugs [13]. This will be expressed as the average number of PRNs/4 h.

### Secondary endpoints

This study will also explore the effects of music on ICU delirium. Delirium is assessed twice a day (per usual care) with the Cornell Assessment of Pediatric Delirium (CAPD) instrument [35]. Those patients with a score > 9 in two consecutive measurements will be considered to have PICU delirium. Vital signs including heart rate, systolic blood pressure, diastolic blood pressure, respiratory rate, and oxygen saturation are being collected prior to the intervention, at 15 min during the intervention, at the end of the intervention, and 30 min after the intervention. This information is being obtained to assess physiologic effects of music in critically ill children and also to monitor adverse effects of this intervention. Other adverse events such as intolerance to the intervention and skin and/or ear problems (e.g., pressure injuries) thought to be associated with the use of headphones are being monitored. Tolerance is being assessed with the use of the SBS as described above.

We are also collecting daily information of possible sources for discomfort or pain including the following: insertion, removal, and/or presence of intravenous lines, arterial line, central venous line, chest tubes, urinary catheter, nasogastric tube, endotracheal tube; dressing changes; sternotomy closure; and/or wound vacuum changes. Duration of invasive mechanical ventilation, PICU stay, and PICU mortality are also being recorded.

## Parents’ survey

As part of our family-centered care approach, we are including parents’ perspective on the use of music for sedation in critically ill children. Parents’ opinions on the intervention are being explored with a survey conducted at the end of the intervention and prior to the patient’s discharge from the ICU (Additional file 2). Parents interested in the study results will be contacted and informed of the study outcomes by email.

## Data handling

Data are being collected using an electronic case report (eCRF) form using REDCap, Research Electronic Data Capture [31]. Monitoring on data collection and consistency checks are being performed by the research coordinator. Original records, including consent, eCRF, and parent’s surveys, will be archived as per local regulations. See Table 1 for the schedule of data collection and interventions.

**Table 1:**
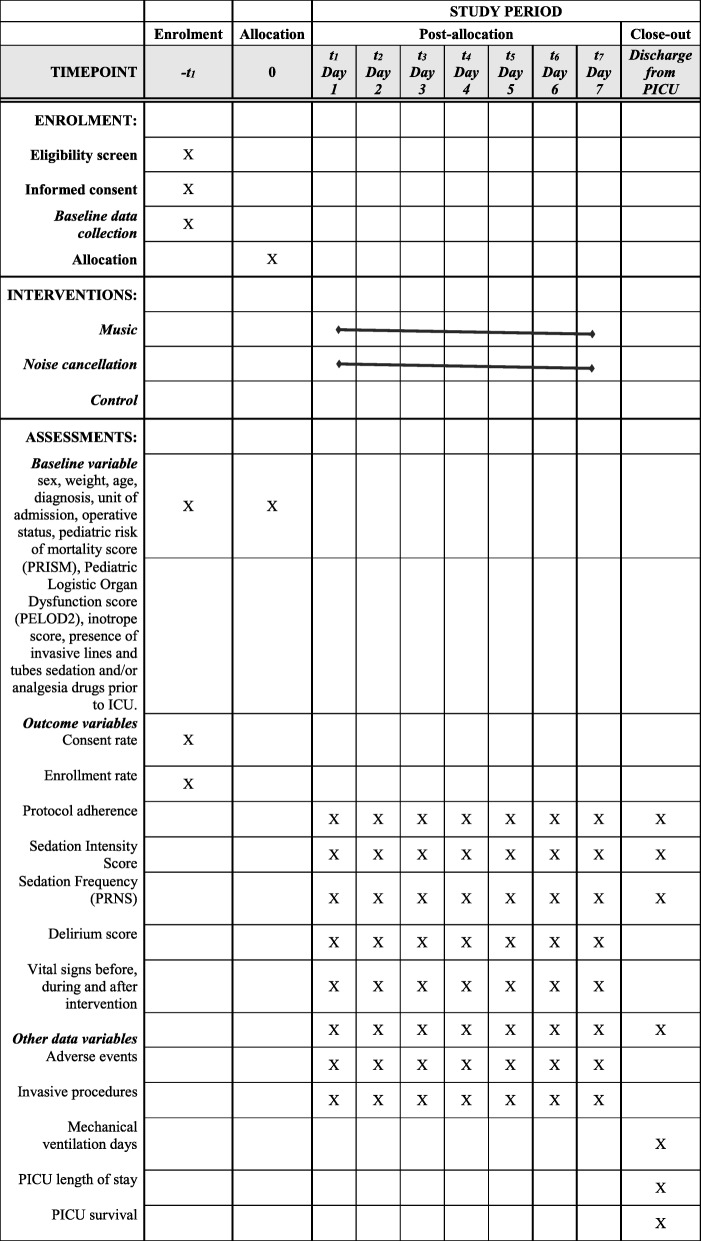
Schedule of enrolment, interventions, and assessments

## Sample size justification

Our primary outcome is protocol adherence. Assuming a protocol acceptance/completion of 80%, 60 patients will be needed to estimate the rate within 10% of the true rate with 95% confidence. Also, this number of participants per group follows the recommended rules for pilot trials’ sample size when the standardized effect size is unknown but expected to be small [36]. With 20 patients in each group, we will obtain pediatric-specific information to calculate a sample size for a future definitive trial.

## Analysis

Baseline characteristics will be presented by descriptive statistics and graphs to show the distribution of the variables. Feasibility outcomes will be presented as percentage and 95% confidence intervals. Analysis of outcomes will be conducted using both intention to treat and per protocol. Linear regression and mixed-effects models will be used to analyze the primary effect of the music on sedation requirements and treatment effect differences between groups. Mixed-effects models will be implemented to accommodate the correlation and inconstant variance between sedation requirement measurements among various time points. Additionally, using mixed-effects models for repeated measurement data analysis will improve the statistical power and decrease biases due to missing data in comparison with using any imputation method which could under-/overestimate treatment effects and standard errors. When feasible, all analysis will be presented with 95% CI to inform the precision of the results. Since the analysis of preliminary pilot data is not usually recommended, this will be preliminary and should be treated with caution. We will use the R version 5.3.0 statistical software for the analysis [37].

## Research ethics approval

Research ethics approval for this study was obtained from the University of Alberta Health Research Ethics Board (Pro00073775). Informed consent is given in writing by the parents or legal guardians after providing study information orally and in writing after admission to the PICU or PCICU (Additional file 3). The study has been registered at ClinicalTrials.gov (NCT03497559).

## Discussion

While music appears to be a promising intervention, there is presently no evidence that it decreases the use of pharmacologic therapies for sedation and analgesia in critically ill children. This pilot study is a necessary first step toward the conduct of a future definitive music trial in critically ill children. In order to design and conduct a larger trial, we need to demonstrate the tolerability and feasibility of a music intervention. This pilot study will also allow formal sample size calculation for a larger trial and will allow us to obtain feedback from major stakeholders, including families.

## Trial status

The study was initiated on March 27, 2018, and finished enrolment on April 11, 2019. We are currently finalizing data collection and we expect to complete the study by June 1, 2019.

## Supplementary information

**Additional file 1.** Data collection case report.

**Additional file 2.** Parents’ opinions on the intervention.

**Additional file 3.** Informed consent.

## Data Availability

Not applicable.

## Abbreviations

CAPD: Cornell assessment of pediatric delirium
ECMO: Extracorporeal membrane oxygenation
eCRF: Electronic case report
EPICORE: Epidemiology Coordinating and Research Centre
ICU: Intensive care unit
PCICU: Pediatric cardiac intensive care unit
PELOD: Pediatric logistic organ dysfunction score
PICU: Pediatric intensive care unit
PRN: Intermittent dose
RCT: Randomized controlled trial
REDCap: Research Electronic Data Capture
SBS: Sedation Behavior Scale
WAT-1: Withdrawal Assessment Tool 1
