# Supplementary material for: Music Use for Sedation in Critically ill Children (MUSiCC trial): study protocol for a pilot randomized controlled trial
Source: Pilot Feasibility Stud. 2020 Feb 25;6:31. doi: 10.1186/s40814-020-0563-x (PMC7043021; doi:10.1186/s40814-020-0563-x)
Supplement: Supplementary file 1 — Additional file 1. Data collection case report. [file 40814_2020_563_MOESM1_ESM.docx]

Pilot randomized controlled trial on Music Use for Sedation In Critically ill Children.

MUSiCC

**
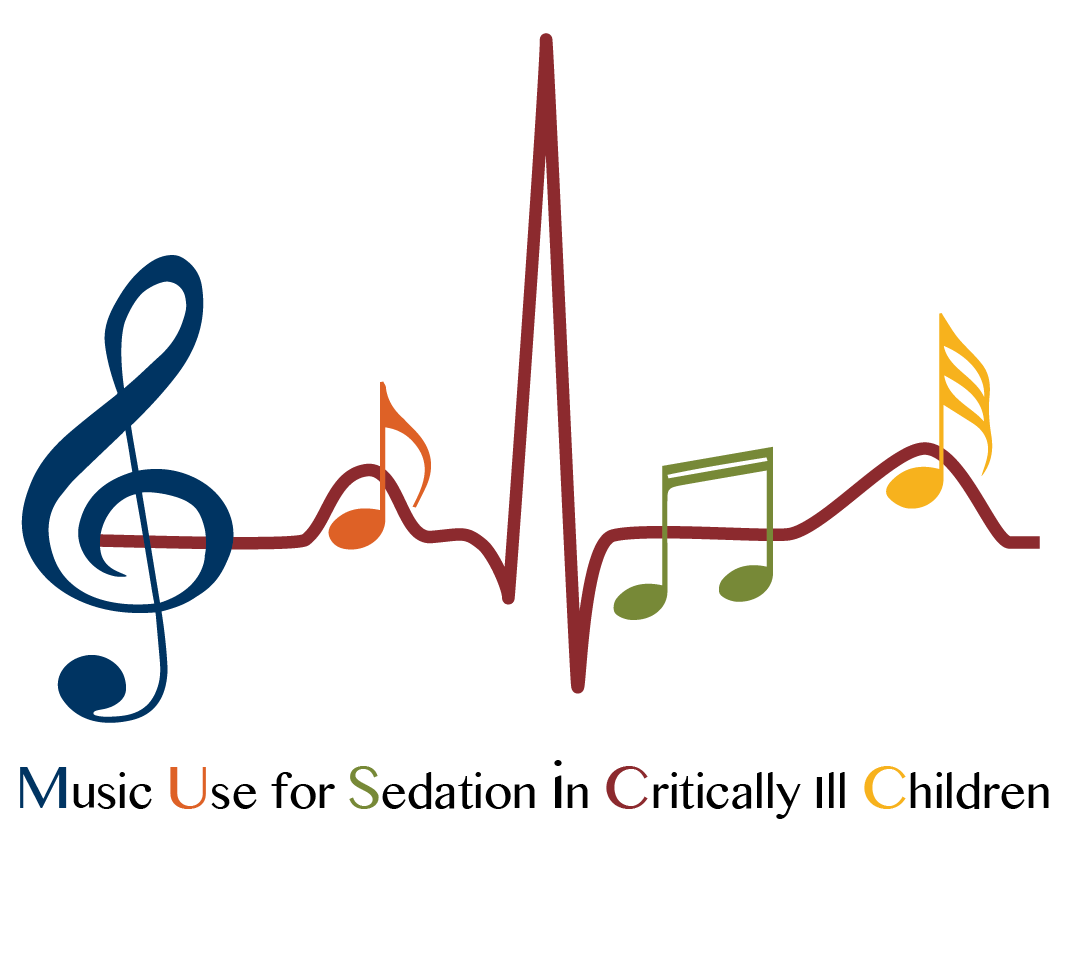
**

**CASE REPORT FORM**

**Patient Information**

*Please complete and return to*:

MUSiCC Project Manager

Stollery Children’s Hospital University of Alberta

**Case Report Forms: Procedure Manual Notes**

**General Instructions**

· At the top of each page, enter the patient study **Number**

· Enter dates in the format dd / mm / yyyy (i.e. October 22, 1998 is 22 / Oct / 1998)

· Enter times according to the 24 hour clock in the format HH:MM (i.e. 4 pm is entered 16:00)

· Do not write in shaded areas

· If data is not applicable, not known, illegible, or incorrect, enter N/A. There should be no blank spaces. If data is missing use the letter M.

· Use only **black ink**

**Source Document**

Complete the information on this page and file it separately in the Patient Source Document Binder.

The document is to be used as reference for patient follow-up. It may also be necessary to access the patient’s medical record in the future for auditing purposes. This information will allow for accurate case identification.

## Source Document

Once this page is completed it has to be detached from the CRF and has to be kept in a separate binder with all the rest of the Source Document forms.

Patient initials: ________________________________

Patient study number: ________________________________

Age in years: *|__|__|*

**FORM 1: INCLUSION/EXCLUSION CRITERIA:**

**1.1 Inclusion Criteria:**

Check “**yes**” for each inclusion criteria present.

**NOTE**: all inclusion criteria must be checked “yes” in order for the patient to be eligible for the study.

**FORM 1. INCLUSION/EXCLUSION CRITERIA**

**1.1 INCLUSION CRITERIA:** (all inclusion criteria must be answered “YES” to include patient)

# YES NO

□ □ 1. Admitted to PICU or PCICU

□ □ 2. Age > 1 months and < 17 years old

□ □ 3. Mechanically ventilated for > 24 hours but not more than 48 hours

□ □ 4. Parental or legal guardian consent

**FORM 1: INCLUSION/EXCLUSION CRITERIA :**

**1.2 Exclusion Criteria:** Check “**no**” for each exclusion criteria NOT present. **NOTE**: all exclusion criteria must be checked “no” in order for the patient to be eligible for the study.

**FORM 1 cont. INCLUSION/EXCLUSION CRITERIA**

**1.2 EXCLUSION CRITERIA (**all exclusion criteria must be answered “NO” to include patient)

**YES** **NO**

□ □ 1. Known hearing deficit

□ □ 2. Major Cranial-facial abnormalities

□ □ 3. Traumatic Brain Injury with suspected high intracranial pressure or GCS ≤10

□ □ 4. Patients receiving paralytic agents

□ □ 5. Patients not expected to survive the next 48 hours

□ □ 6. Non-invasive mechanical ventilation

□ □ 7. Infants < 1 months of age and/or unable to fit the headphones

□ □ 8. Patients not receiving sedation and/or analgesia drugs

□ □ 9. Patients enrolled in another sedation intervention study.

**Form 2 – ADMISSION AND DEMOGRAPHICS:**

**2-1 Sex**: Check either male or female

**2-2 Weight on admission**: This is the weight at admission in Kg with one decimal.

**2-3 Age on admission**: Age in years (If less than 1 year, divide the number of months by 12).

**2-4 Pediatric Risk of Mortality Score (PRISM)**

**2-5 Admission to PCICU or PICU**: Record if the patient was admitted under PCICU or PICU team. Please note that this may not exactly coincide with physical unit location: for example, a PICU team patient may be cared for by the PICU team in the PCICU location.

**2-6 Sedation and/or analgesia drugs pre-admission to ICU**: State if the patient was receiving sedation/analgesia drugs prior to the admission to PICU or PCICU. This does not include anesthetics for a surgical procedure just prior to the admission. If the answer is “Yes” specify the number of days the patient was on sedation or analgesia medications.

**2-7 Admission post-operatively**: Please record if the patient was admitted to ICU after a surgical procedure.

**2-8 Cardiac diagnosis:** Please check “Yes” if the patient has a congenital and/or acquired heart disease. Check “No” otherwise.

**2-9 Diagnosis:** Please report the patient diagnosis on admission. **Use the following categories for diagnosis**:

1. Post-operative Patient recovering from surgical procedure (non-cardiac): 1a. general surgery, 1b. neurosurgery, or 1c. ENT

2. Shock: 2a. Septic shock, 2b. hypovolemic shock (dehydration, bleeding), 2c. vasodilatory shock (anaphylaxis, overdose, spinal trauma)

3. Respiratory: Respiratory distress/failure (3a. pneumonia, 3b. bronchiolitis, 3c. aspiration, 3d. acute respiratory distress syndrome, 3e. other, e.g., croup)

4. Gastrointestinal: Gastrointestinal 4a. bleed, or 4b. liver failure, or 4c. liver transplant

5. Neurologic: Coma, seizures, encephalitis, meningitis, encephalopathy, Guillain-Barre syndrome, Asphyxia (e.g., hanging)

6. Trauma: 6a. Multitrauma [e.g., liver or spleen injury, bowel injury, fractures], 6b. Traumatic brain injury, 6c. Burn

7. Cardiac arrest Admitted after a cardiac arrest

8. Renal failure Admitted for primary kidney disease needing dialysis

9. Other If not captured in above

**2-10 Admission to Stollery Children’s PICU/PCICU date and time**: This is the date and time the patient arrives at the Stollery PICU/PCICU.

**2-11 Date and time of enrolment**: Date and time when the patient was included in the study.

**2-12 Arterial line:** Please check “Yes” if the patient has an arterial line at the time of enrollment

**2-13 Central line:** Please check “Yes” if the patient has a central line (including PICC lines) at the time of enrollment

**2-14 Chest tube:** Please check “Yes” if the patient has a chest tube at the time of enrollment

**2-15 Mediastinal tube:** Please check “Yes” if the patient has a mediastinal tube at the time of enrollment

**2-16 Inotrope score:** record the highest inotrope score for the day of enrolment. Inotrope Score is calculated as follows: Dopamine dose (μg/kg/min) + Dobutamine dose (μg/kg/min) +100 × epinephrine dose (μg/kg/min) + 100 X norepinephrine dose (ug/kg/min) + 10 X Milrinone dose (μg/kg/min) +10,000 × Vasopressin dose (U/kg/min).

**2-17 PELOD2 score:** record the Pelod score for the day of enrollment. For PELOD scoring consider the following (PELOD scoring tool at the end of the CRF):

a. GCS is that expected without sedation. Only can be abnormal in patients with known or suspected central nervous system disease.

-If a GCS lower than 11 is not due to a brain injury [e.g., not admitted for cardiac arrest, meningitis, head trauma, neurosurgery, seizures, stroke, intracranial bleeding, hydrocephalus] or not due to a chronic brain dysfunction [e.g., not severe developmental delay], then record GCS as normal.

-In intubated patients, sometimes the GCS is scored out of 10 because the patient cannot verbally make sounds with the ETT in. Thus, a GCS of 10/10 is normal.

-check the MD Notes: if when giving CNS note, there is no mention of a concern about level of consciousness, response to stimulus, or seizures, then the GCS is considered normal.

b. Pupils: nonreactive pupils must be >3mm in size to record as nonreactive on PELOD.

c. Pa02 is only on arterial blood gas [not from venous or capillary blood gases].

-if there is no arterial blood gas, then Pa02 is assumed normal.

-PaC02 can be from arterial, venous, or capillary blood gas.

d. Can use worst values for creatinine, WBC, and platelets from 24hr pre- to 4hr post- calorimetry.

-if not measured, then assume these values are normal.

**FORM 2. BASELINE AND DEMOGRAPHICS**

**2-1. Sex:** *|__| Male |__| Female*

**2-2. Weight on admission:** |__|__|.|__| Kg

**2-3. Age on admission:** |__|__| years

**2-4. PRISM:** |__|__|

**2-5. Intensivist team caring for the patient:** PCICU |__| PICU |__|

**2-6. Sedation and/or analgesia drugs pre-admission to PICU/PCICU:**  Yes |__| No |__| If yes, how many days |__|__|

**2-7. Admission post-operatively:** Yes |__| No |__|

**2-8. Cardiac diagnosis:** Yes |__| No |__|

**2-9. Diagnosis on admission:** *|__|__|*

**2-10. Admission to Stollery Children’s Hospital PICU/PCICU date:***|__|__| |__|__|__| |__|__|__|__|*

*D D / M M M / Y E A R*

**Admission to Stollery Children’s Hospital PICU/PCICU time:** *|__|__|HH* ***:*** *|__|__| MIN*

**2-11 Enrolment date:***|__|__| |__|__|__| |__|__|__|__|*

*D D / M M M / Y E A R*

**Enrolment time:** *|__|__|HH* ***:*** *|__|__| MIN*

**2-12 Arterial line:** Yes |__| No |__|

**2-13 Central line:** Yes |__| No |__|

**2-14 Chest tube:** Yes |__| No |__|

**2-15 Mediastinal tube:** Yes |__| No |__|

**2-16 Inotrope score at enrolment:** *|__|__|*

**2-17 PELOD 2 at enrolment:** *|__|__|*

………………………………………………………………………

**FORM 3: Sedation and analgesia daily information.**

This should be recorded for every day the patient was ventilated in the ICU and while the study is being conducted (maximum of 7 days, enrollment date is day 1; any part of a day is recorded as one day, and days are according to our charting from 0700 to 0659 hrs [e.g., if admitted at 2300 on May 1, then May 1 is day 1, May 2 is day 2 starting at 0700hrs).

**3-0. Date:** This is the date of the patient stay (i.e. If we are collecting information of the sedation the patient received on March 12^th^, we should enter 12/Mar/2017)

**3-1. Continuous sedation and analgesia drugs:** Enter the name and dose per kilogram of all the sedation and analgesia drugs the patient received as a continuous infusion in each 4 hour block for that day. For example: if the patient is receiving at 7:00h a morphine infusion at 30 mcg/kg/hour and at 9:00h the dose is changed to 40 mcg/kg/hour, the dose enter for the 7am -11am block is = 30 x 2 + 40 x 2 = 140 mcg/kg.

**3-2. Sedation and analgesia intermittent and PRNs doses given:** Enter the drug, dose per kilogram and the time when these doses were given, and if they were given for a particular procedure (intubation, IV start, CVL, arterial line, dressing change, chest closure, chest tube removal, etc).

**3-3. Sedation/pain scores:** Enter the type of score performed, the number scored and the time of the scoring. State Behavior Scale: +2 to -3. Goal is usually 0 to -1. FLACC: 0 to 10. Score >3 needs treatment. In non-ventilated children can use Faces or Numeric score instead; if so, record the value in the FLACC entry space. If a score is not recorded, check: “not done”.

**3-4. Withdrawal scores:** Enter the type of score performed, the number scored and the time of the scoring. WAT-1: 0 to 12. Score >3 indicates possible withdrawal. If a score is not recorded, check: “not done”.

**3-5. Delirium scores:** Enter the type of score performed, the number scored and the time of the scoring. CAP-D: 0 to 32. Score >9 indicates delirium. If a score is not recorded, check: “not done”.

**FORM 3. Sedation and analgesia daily information**

**Day** *|__|*

**3-0. Date:** *|__|__| |__|__|__| |__|__|__|__|*

D D / M M M /Y E A R

**3-1. Continuous sedation and analgesia drugs:**

**Dose of Morphine: 07:00 to 11:00** |__|__|.|__|__| mcg/kg

**11:00 to 15:00**  |__|__|.|__|__| mcg/kg

**15:00 to 19:00**  |__|__|.|__|__| mcg/kg

**19:00 to 23:00** |__|__|.|__|__| mcg/kg

**23:00 to 03:00** |__|__|.|__|__| mcg/kg

**03:00 to 07:00**  |__|__|.|__|__| mcg/kg

**Dose of Midazolam: 07:00 to 11:00** |__|__|.|__|__| mcg/kg

**11:00 to 15:00**  |__|__|.|__|__| mcg/kg

**15:00 to 19:00**  |__|__|.|__|__| mcg/kg

**19:00 to 23:00** |__|__|.|__|__| mcg/kg

**23:00 to 03:00** |__|__|.|__|__| mcg/kg

**03:00 to 07:00**  |__|__|.|__|__| mcg/kg

**Dose of Hydromorphone: 07:00 to 11:00** |__|__|.|__|__| mcg/kg

**11:00 to 15:00**  |__|__|.|__|__| mcg/kg

**15:00 to 19:00**  |__|__|.|__|__| mcg/kg

**19:00 to 23:00** |__|__|.|__|__| mcg/kg

**23:00 to 03:00** |__|__|.|__|__| mcg/kg

**03:00 to 07:00**  |__|__|.|__|__| mcg/kg

**Dose of Dexmedetomidine:** **07:00 to 11:00** |__|__|.|__|__| mcg/kg

**11:00 to 15:00**  |__|__|.|__|__| mcg/kg

**15:00 to 19:00**  |__|__|.|__|__| mcg/kg

**19:00 to 23:00** |__|__|.|__|__| mcg/kg

**23:00 to 03:00** |__|__|.|__|__| mcg/kg

**03:00 to 07:00**  |__|__|.|__|__| mcg/kg

**Dose of Propofol:** **07:00 to 11:00** |__|__|.|__|__| mg/kg

**11:00 to 15:00**  |__|__|.|__|__| mg/kg

**15:00 to 19:00**  |__|__|.|__|__| mg/kg

**19:00 to 23:00** |__|__|.|__|__| mg/kg

**23:00 to 03:00** |__|__|.|__|__| mg/kg

**03:00 to 07:00**  |__|__|.|__|__| mg/kg

**Dose of Fentanyl:** **07:00 to 11:00** |__|__|.|__|__| mcg/kg

**11:00 to 15:00**  |__|__|.|__|__| mcg/kg

**15:00 to 19:00**  |__|__|.|__|__| mcg/kg

**19:00 to 23:00** |__|__|.|__|__| mcg/kg

**23:00 to 03:00** |__|__|.|__|__| mcg/kg

**03:00 to 07:00**  |__|__|.|__|__| mcg/kg

**Study day** *|__|*

**Date:** *|__|__| |__|__|__| |__|__|__|__|*

D D / M M M /Y E A R

**Dose of ketamine:** **07:00 to 11:00** |__|__|.|__|__| mcg/kg

**11:00 to 15:00**  |__|__|.|__|__| mcg/kg

**15:00 to 19:00**  |__|__|.|__|__| mcg/kg

**19:00 to 23:00** |__|__|.|__|__| mcg/kg

**23:00 to 03:00** |__|__|.|__|__| mcg/kg

**03:00 to 07:00**  |__|__|.|__|__| mcg/kg

Other:…………………(name) **07:00 to 11:00** |__|__|.|__|__| **___**/kg

**11:00 to 15:00**  |__|__|.|__|__| ___/kg

**15:00 to 19:00**  |__|__|.|__|__| ___/kg

**19:00 to 23:00** |__|__|.|__|__| ___/kg

**23:00 to 03:00** |__|__|.|__|__| ___/kg

**03:00 to 07:00**  |__|__|.|__|__| ___/kg

**3-2. Sedation and analgesia intermittent (PRNs) doses given:**

**Morphine:**

**07:00 to 11:00 Number doses:** |__|__| **Cumulative dose**|__|__|.|__|__| mg/kg

**11:00 to 15:00 Number doses:** |__|__| **Cumulative dose**|__|__|.|__|__| mg/kg

**15:00 to 19:00 Number doses:** |__|__| **Cumulative dose**|__|__|.|__|__| mg/kg

**19:00 to 23:00** **Number doses:** |__|__| **Cumulative dose**|__|__|.|__|__| mg/kg

**23:00 to 03:00** **Number doses:** |__|__| **Cumulative dose**|__|__|.|__|__| mg/kg

**03:00 to 07:00 Number doses:** |__|__| **Cumulative dose**|__|__|.|__|__| mg/kg

**Lorazepam:**

**07:00 to 11:00 Number doses:** |__|__| **Cumulative dose**|__|__|.|__|__| mg/kg

**11:00 to 15:00 Number doses:** |__|__| **Cumulative dose**|__|__|.|__|__| mg/kg

**15:00 to 19:00 Number doses:** |__|__| **Cumulative dose**|__|__|.|__|__| mg/kg

**19:00 to 23:00** **Number doses:** |__|__| **Cumulative dose**|__|__|.|__|__| mg/kg

**23:00 to 03:00** **Number doses:** |__|__| **Cumulative dose**|__|__|.|__|__| mg/kg

**03:00 to 07:00 Number doses:** |__|__| **Cumulative dose**|__|__|.|__|__| mg/kg

**Chloral hydrate:**

**07:00 to 11:00 Number doses:** |__|__| **Cumulative dose**|__|__|.|__|__| mg/kg

**11:00 to 15:00 Number doses:** |__|__| **Cumulative dose**|__|__|.|__|__| mg/kg

**15:00 to 19:00 Number doses:** |__|__| **Cumulative dose**|__|__|.|__|__| mg/kg

**19:00 to 23:00** **Number doses:** |__|__| **Cumulative dose**|__|__|.|__|__| mg/kg

**23:00 to 03:00** **Number doses:** |__|__| **Cumulative dose**|__|__|.|__|__| mg/kg

**03:00 to 07:00 Number doses:** |__|__| **Cumulative dose**|__|__|.|__|__| mg/kg

**Study day** *|__|*

**Date:** *|__|__| |__|__|__| |__|__|__|__|*

D D / M M M /Y E A R

**Clonidine:**

**07:00 to 11:00 Number doses:** |__|__| **Cumulative dose**|__|__|.|__|__| mg/kg

**11:00 to 15:00 Number doses:** |__|__| **Cumulative dose**|__|__|.|__|__| mg/kg

**15:00 to 19:00 Number doses:** |__|__| **Cumulative dose**|__|__|.|__|__| mg/kg

**19:00 to 23:00** **Number doses:** |__|__| **Cumulative dose**|__|__|.|__|__| mg/kg

**23:00 to 03:00** **Number doses:** |__|__| **Cumulative dose**|__|__|.|__|__| mg/kg

**03:00 to 07:00 Number doses:** |__|__| **Cumulative dose**|__|__|.|__|__| mg/kg

**Propofol:**

**07:00 to 11:00 Number doses:** |__|__| **Cumulative dose**|__|__|.|__|__| mg/kg

**11:00 to 15:00 Number doses:** |__|__| **Cumulative dose**|__|__|.|__|__| mg/kg

**15:00 to 19:00 Number doses:** |__|__| **Cumulative dose**|__|__|.|__|__| mg/kg

**19:00 to 23:00** **Number doses:** |__|__| **Cumulative dose**|__|__|.|__|__| mg/kg

**23:00 to 03:00** **Number doses:** |__|__| **Cumulative dose**|__|__|.|__|__| mg/kg

**03:00 to 07:00 Number doses:** |__|__| **Cumulative dose**|__|__|.|__|__| mg/kg

**Fentanyl:**

**07:00 to 11:00 Number doses:** |__|__| **Cumulative dose**|__|__|.|__|__| mg/kg

**11:00 to 15:00 Number doses:** |__|__| **Cumulative dose**|__|__|.|__|__| mg/kg

**15:00 to 19:00 Number doses:** |__|__| **Cumulative dose**|__|__|.|__|__| mg/kg

**19:00 to 23:00** **Number doses:** |__|__| **Cumulative dose**|__|__|.|__|__| mg/kg

**23:00 to 03:00** **Number doses:** |__|__| **Cumulative dose**|__|__|.|__|__| mg/kg

**03:00 to 07:00 Number doses:** |__|__| **Cumulative dose**|__|__|.|__|__| mg/kg

**Ketamine:**

**07:00 to 11:00 Number doses:** |__|__| **Cumulative dose**|__|__|.|__|__| mg/kg

**11:00 to 15:00 Number doses:** |__|__| **Cumulative dose**|__|__|.|__|__| mg/kg

**15:00 to 19:00 Number doses:** |__|__| **Cumulative dose**|__|__|.|__|__| mg/kg

**19:00 to 23:00** **Number doses:** |__|__| **Cumulative dose**|__|__|.|__|__| mg/kg

**23:00 to 03:00** **Number doses:** |__|__| **Cumulative dose**|__|__|.|__|__| mg/kg

**03:00 to 07:00 Number doses:** |__|__| **Cumulative dose**|__|__|.|__|__| mg/kg

**Other:**…………………

Name

**07:00 to 11:00 Number doses:** |__|__| **Cumulative dose**|__|__|.|__|__| mg/kg

**11:00 to 15:00 Number doses:** |__|__| **Cumulative dose**|__|__|.|__|__| mg/kg

**15:00 to 19:00 Number doses:** |__|__| **Cumulative dose**|__|__|.|__|__| mg/kg

**19:00 to 23:00** **Number doses:** |__|__| **Cumulative dose**|__|__|.|__|__| mg/kg

**23:00 to 03:00** **Number doses:** |__|__| **Cumulative dose**|__|__|.|__|__| mg/kg

**03:00 to 07:00 Number doses:** |__|__| **Cumulative dose**|__|__|.|__|__| mg/kg

**3-3. Sedation scores:**

|__| **SBS:** |__|__| score Time scored:|__|__|.|__|__| Hs.

|__|__| score Time scored:|__|__|.|__|__| Hs.

|__|__| score Time scored:|__|__|.|__|__| Hs.

|__|__| score Time scored:|__|__|.|__|__| Hs.

Not done |__|

|__| **FLACC:** |__|__| score Time scored:|__|__|.|__|__| Hs.

|__|__| score Time scored:|__|__|.|__|__| Hs.

|__|__| score Time scored:|__|__|.|__|__| Hs.

|__|__| score Time scored:|__|__|.|__|__| Hs.

Not done |__|

**3-4. Withdrawal scores:**

|__| **WAT-1:** |__|__| score Time scored:|__|__|.|__|__| H

|__|__| score Time scored:|__|__|.|__|__| H

|__|__| score Time scored:|__|__|.|__|__| H

|__|__| score Time scored:|__|__|.|__|__| H

Not done |__|

**3-5. Delirium scores:**

|__| **CAPD:** |__|__| score Time scored:|__|__|.|__|__| H

|__|__| score Time scored:|__|__|.|__|__| H

|__|__| score Time scored:|__|__|.|__|__| H

|__|__| score Time scored:|__|__|.|__|__| H

Not done |__|

**FORM 4: Intervention daily information:**

This should be recorded for every day the patient stays ventilated in the ICU and while the study was being conducted (maximum of 7 days since enrollment).

**4-0. Date:** This is the date of the patient stay (i.e. If we are collecting information of the sedation the patient received on March 12^th^, we should enter 12/Mar/2017)

**4.1 Time of intervention:** The corresponding intervention should be performed for 30 minutes 3 times a day: morning (M) between 07:00h and 12:00h, afternoon (A) between 12:00h and 16:00h, and evening (E) between 16:00h and 20:00h. The bedside nurse will decide the exact time of the intervention based on the patient status and procedures. Each intervention will take place for a minimum 30 minutes. For each intervention please record:

1. Time: Record the time when the intervention was started and discontinued during that day. Write N/A if patient was in the control arm.
2. Duration (in minutes)
3. Discontinuation: If the intervention was stopped prior to 30 minutes, please record the reason.

**4.2 Heart rate:** Record the HR

a) Prior to the intervention

b) 15 minutes after the start of the intervention

c) At the end of the intervention

d) 30 minutes after the completion of the intervention

**4.3 Respiratory rate:** Record the RR

a) Prior to the intervention

b) 15 minutes after the start of the intervention

c) At the end of the intervention

d) 30 minutes after the completion of the intervention

**4.4 Systolic Blood pressure:** Record SBP only if patient has an arterial line

a) Prior to the intervention

b) 15 minutes after the start of the intervention

c) At the end of the intervention

d) 30 minutes after the completion of the intervention

**4.5 Diastolic Blood pressure:** Record DBP only if patient has an arterial line

a) Prior to the intervention

b) 15 minutes after the start of the intervention

c) At the end of the intervention

d) 30 minutes after the completion of the intervention

**4.6 Oxygen Saturation:** Record O2Sats

a) Prior to the intervention

b) 15 minutes after the start of the intervention

c) At the end of the intervention

d) 30 minutes after the completion of the intervention

***FORM 4:* Daily Information*:***

**Study Day** *|__|*

**4.0 Date:** *|__|__| |__|__|__| |__|__|__|__|*

D D / M M M /Y E A R

**4.1M Intervention**

**Morning (M):** Done? Yes |__| No |__| If no, why______________________?

1. **Time started**:|__|__|.|__|__| H **Time stopped**:|__|__|.|__|__| H
2. **Duration** |__|__| minutes
3. **Discontinuation prior to 30 minutes**

No |__| Yes |__| If yes, why: |__| Patient in OR |__| Patient too unstable

|__| Procedure being done to the patient |__| Family asked not to do it

|__| Patient gone for CT or other diagnostic test/intervention

Other ______________________

**4.2.M Heart Rate:**

a) Prior to the intervention *|__|__|__|*

b) At 15 minutes of the intervention *|__|__|__|*

c) At the end of the intervention *|__|__|__|*

d) 30 minutes after the intervention *|__|__|__|*

**4.3.M Respiratory rate:**

a) Prior to the intervention *|__|__|*

b) At 15 minutes of the intervention *|__|__|*

c) At the end of the intervention *|__|__|*

d) 30 minutes after the intervention *|__|__|*

**4.4.M Systolic Blood pressure:**

a) Prior to the intervention *|__|__|__|*

b) At 15 minutes of the intervention *|__|__|__|*

c) At the end of the intervention *|__|__|__|*

d) 30 minutes after the intervention *|__|__|__|*

**4.5.M Diastolic Blood pressure**

a) Prior to the intervention *|__|__|*

b) At 15 minutes of the intervention *|__|__|*

c) At the end of the intervention *|__|__|*

d) 30 minutes after the intervention *|__|__|*

**4.6.M Oxygen Saturation**

a) Prior to the intervention *|__|__|__|*

b) At 15 minutes of the intervention *|__|__|__|*

c) At the end of the intervention *|__|__|__|*

d) 30 minutes after the intervention *|__|__|__|*

***FORM 4:* Daily Information*:***

**Study Day** *|__|*

**4.0 Date:** *|__|__| |__|__|__| |__|__|__|__|*

D D / M M M /Y E A R

**4.1A Intervention**

**Afternoon (A):** Done? Yes |__| No |__| If no, why______________________?

1. **Time started**:|__|__|.|__|__| H **Time stopped**:|__|__|.|__|__| H
2. **Duration** |__|__| minutes
3. **Discontinuation prior to 30 minutes** No |__| Yes |__| If yes, why______________________?

**4.2.A Heart Rate:**

a) Prior to the intervention *|__|__|__|*

b) At 15 minutes of the intervention *|__|__|__|*

c) At the end of the intervention *|__|__|__|*

d) 30 minutes after the intervention *|__|__|__|*

**4.3.A Respiratory rate:**

a) Prior to the intervention *|__|__|*

b) At 15 minutes of the intervention *|__|__|*

c) At the end of the intervention *|__|__|*

d) 30 minutes after the intervention *|__|__|*

**4.4.A Systolic Blood pressure:**

a) Prior to the intervention *|__|__|__|*

b) At 15 minutes of the intervention *|__|__|__|*

c) At the end of the intervention *|__|__|__|*

d) 30 minutes after the intervention *|__|__|__|*

**4.5.A Diastolic Blood pressure**

a) Prior to the intervention *|__|__|*

b) At 15 minutes of the intervention *|__|__|*

c) At the end of the intervention *|__|__|*

d) 30 minutes after the intervention *|__|__|*

**4.6.A Oxygen Saturation**

a) Prior to the intervention *|__|__|__|*

b) At 15 minutes of the intervention *|__|__|__|*

c) At the end of the intervention *|__|__|__|*

d) 30 minutes after the intervention *|__|__|__|*

***FORM 4:* Daily Information*:***

**Study Day 1**

**4.0 Date:** *|__|__| |__|__|__| |__|__|__|__|*

D D / M M M /Y E A R

**4.1E Intervention**

**Evening (E):** Done? Yes |__| No |__| If no, why______________________?

1. **Time started**:|__|__|.|__|__| H **Time stopped**:|__|__|.|__|__| H
2. **Duration** |__|__| minutes
3. **Discontinuation prior to 30 minutes** No |__| Yes |__| If yes, why______________________?

**4.2.E Heart Rate:**

a) Prior to the intervention *|__|__|__|*

b) At 15 minutes of the intervention *|__|__|__|*

c) At the end of the intervention *|__|__|__|*

d) 30 minutes after the intervention *|__|__|__|*

**4.3.E Respiratory rate:**

a) Prior to the intervention *|__|__|*

b) At 15 minutes of the intervention *|__|__|*

c) At the end of the intervention *|__|__|*

d) 30 minutes after the intervention *|__|__|*

**4.4.E Systolic Blood pressure:**

a) Prior to the intervention *|__|__|__|*

b) At 15 minutes of the intervention *|__|__|__|*

c) At the end of the intervention *|__|__|__|*

d) 30 minutes after the intervention *|__|__|__|*

**4.5.E Diastolic Blood pressure**

a) Prior to the intervention *|__|__|*

b) At 15 minutes of the intervention *|__|__|*

c) At the end of the intervention *|__|__|*

d) 30 minutes after the intervention *|__|__|*

**4.6.E Oxygen Saturation**

a) Prior to the intervention *|__|__|__|*

b) At 15 minutes of the intervention *|__|__|__|*

c) At the end of the intervention *|__|__|__|*

d) 30 minutes after the intervention *|__|__|__|*

***FORM 5:* Other daily Information*:***

**5.1 Procedures:** record which procedures and at what time were conducted that day. Procedures include: intubation, central venous line insertion, arterial line insertion, chest tube insertion (including mediastinal tubes), chest tube removal (including mediastinal tubes), nasogastric(NG) or nasojejunal(NJ) tubes, foley catheter, dressing changes, wound vacuum changes, intubation, IV insertion or other major procedures that required sedation within the unit.

**5.2 Major sources of discomfort:** check if the patient has already any of the following in place: central venous line, arterial line, chest tube (including mediastinal tubes), Foley catheter.

***FORM 5:* Other daily Information*:***

**5.1 Procedures:** Central venous line |__| Arterial line|__| Intubation|__|

Chest tube insertion|__| Chest tube removal|__| NG/NJ insertion|__|

Foley catheter|__| Dressing change|__| Wound vacuum|__|

Intubation |__| IV Insertion|__| Other:………….. |__|

**5.2 Major source discomfort:** Central venous line |__| Arterial line|__| Endotracheal tube|__|

Chest tube|__| NG/NJ insertion|__| Foley catheter|__|

Other:………….. |__|

**FORM 6: MORBIDITY AND MORTALITY:**

**6-1 MORBIDITY:**

**6-1.1** **Date invasive mechanical ventilation discontinued:** Record the date according to the date format listed. This is defined as the date the patient is able to breathe spontaneously with no mechanical ventilation or endotracheal tube (with or without tracheostomy). The patient must remain spontaneously breathing for at least 24 hours without reintubation to satisfy this endpoint.

**6-1.2 Length of mechanical ventilation:** Record total number of days of mechanical ventilation during PICU stay after surgery. **Note:** Any portion of a day is considered a full day. For example, a patient is intubated at 2200h on Jan 10^th^ (day 1) and extubated on January 17^th^ (day 8). The length of mechanical ventilation is 8 days.

**6-1.3 Date of discharge from PICU:** Record the date according to the date format listed above. Transfer to NICU or another hospital would be considered discharge from PICU/PCICU.

**6-1.4 Length of PICU/PCICU stay:** Record total number of days in PICU after admission. **Note:** Any portion of a day is considered a full day. One day extends from 0700 until 0659 the following day.

**FORM 6: MORBIDITY, MORTALITY:**

**6-1 MORBIDITY**

**6-1.1 Date invasive mechanical ventilation discontinued:** |__|__| |__|__|__| |__|__|__|__|

D D / M M M / Y Y Y Y

**6-1.2 Total duration of mechanical ventilation during PICU/PCICU stay:** |__|__|__| days

**6-1.3 Date of discharge from PICU/PCICU**: |__|__| |__|__|__| |__|__|__|__|

D D / M M M / Y Y Y Y

**6-1.4 Total length of PICU/PCICU stay since admission:** |_____| days

**6-2 MORBIDITY, MORTALITY CONT**

**6-2.1 PICU/PCICU Survival Status:** Check yes or no to indicate if the patient survived to PICU/PCICU discharge

**6-2.2 Date of death**: Record this in the date format specified.

**6-2.3 Number of days from inclusion to death**: record number of days from inclusion to death.

**6-2.4 Cause of Death**: Select the primary cause of death. Please discuss the most probable cause of death with the attending physician. You may select more than one cause.

Established MODS = MODS occurring and persisting after 48 hours of presentation of shock.

Refractory shock = Hypotension not responding to maximum medical therapy.

Dysrrhythmia = Heart rhythm other than sinus that because it is too fast or too low leads to death

Neurologic sequelae = severe brain injury.

**FORM 6: MORBIDITY, MORTALITY (cont.)**

**6-2 MORTALITY:**

**6-2.1 Survival to discharge from PICU** *|__| Yes |__| No*

**6-2.2 Date of death** *|__|__| |__|__| |__|__*|*__|__|*

*D D / M M / Y Y Y Y*

**6-2.3 Number of days from inclusion to death** *|_*___*_| days*

**6-2.4 Cause of death**

Refractory Shock *|__| yes |__| no*

Established MODS *|__| yes |__| no*

Severe myocardial dysfunction *|__| yes |__| no*

Dysrhythmia *|__| yes |__| no*

Withdrawal of life sustaining therapy *|__| yes |__| no*

Other(s): ________________________ *|__| yes |__| no*

**FORM 7: WITHDRAWAL FROM THE STUDY:**

**WITHDRAWAL FROM THE STUDY:** Check "yes" if the patient has been withdrawn from the study within 7 days after the randomization, and check the appropriate reason.

**FORM 7: WITHDRAWL FROM THE STUDY**

| **Withdrawal from study** | \|__\| yes \|__\| no |
| --- | --- |
| **If yes**, check the appropriate reason | |
| **7-1** Parents asked to withdraw the child from the trial:  (*Justification:____________________________________* ) | \|__\| yes \|__\| no |
| **7-2** Physician asked to withdraw the child from the trial:  (*Justification:________________________________* ) | \|__\| yes \|__\| no |
| **7-3** Other cause of withdrawal:  (*Specify:*_______________________________________ ) | \|__\| yes \|__\| no |
| **7-4** Date of withdrawal | *\|__\|__\| \|__\|__\|__\| \|__\|__ \|__\|__\|*  *DD M M M Y Y Y Y* |

**FORM 8: SUSPENSION OF COMPLIANCE WITH PROTOCOL:**

**8.1 WAS THERE SUSPENSION OF COMPLIANCE WITH THE STUDY PROTOCOL?**

If there was a suspension with compliance with the study protocol, check yes.

Suspension of compliance is defined as not receiving the allocated intervention 2 times or more per day.

- 1. **8.2 Suspected intervention related adverse event**

If compliance with the study protocol is suspended because of a suspected adverse event, mark “yes” and specify reason in form 8.

**8.3 Other**

If the suspension was not suspected to be because of an intervention related adverse event, specify the reason.

**8.4 Date of suspension of compliance to study protocol.**

Record the date according to the date format listed above

**FORM 8: SUSPENSION OF COMPLIANCE WITH PROTOCOL:**

| **Was there a suspension of compliance with study protocol?** | *\|__\| yes \|__\| no* |
| --- | --- |
| ***If yes****, check the appropriate reason(s)* | |
| 8-1 Suspected study intervention related adverse event *(see form on page 46)*: | *\|__\| yes \|__\| no* |
| 8-2 Other – specify:____________________________________ | *\|__\| yes \|__\| no* |
| 8-3 Date of suspension of compliance to study protocol: | *\|__\|__\| \|__\|__\|__\| \|__\|__ \|__\|__\|*  *DD M M M Y Y Y Y* |

**FORM 9: SERIOUS ADVERSE EVENTS:**

**Serious Adverse Event Information:**

Record all serious adverse events that occurred to the patient from the date of admission, until the *first* of the following events: death, PICU discharge, 7 days after enrolment or successful extubation.

**Date and Time:** Using the most applicable units (i.e. minutes, hours, days) indicate the time that the adverse event began.

**Duration:** Using the most applicable units (i.e. minutes, hours, days) indicate the duration of the adverse event.

**Intervention Relationship:** Indicate with the appropriate number the degree of relationship of the intervention to the adverse event according to the scale provided. **Please review with the Site Investigator/attending intensivist.**

**Treatment Required:** Indicate with the appropriate number the treatment required for the adverse event according to the scale provided, more than one option may be selected.

**Patient Outcome:** Indicate with the appropriate number, the outcome of the adverse event according to the scale provided.

**Date of resolution**: Record the date that the adverse event stopped, or was resolved. If the patient died, enter the date of death. **Please review with the site investigator or the attending intensivist.**

**Definition of a Serious Adverse Event (SAE)**

An event is considered “serious” if any of the following situations occur:

- Death
- Life-threatening: refers to an event in which the patient was, in the view of the investigator, at risk of death from the event if medical intervention had not occurred.
- Prolonged Patient In-hospitalization: if a complication prolongs a patient’s hospitalization, the event is considered “serious”.
- Resulting in Persistent or Significant Disability/Incapacity: a substantial disruption of a person’s ability to conduct normal life functions.
- Other medically important condition (specify)

**FORM 9: SERIOUS ADVERSE EVENTS:**

**Serious Adverse Event Information**

| **Description of event** | **Date**  (dd/mmm/yyyy)  **Time**  (H H) | **Duration**  **(mins, hours)** | **Study intervention relationship**  1 = none  2 = possible  3 = probable  4 = definite  5 = insufficient data | **Treatment required (enter all that apply)**  1 = none  2 = medication  3 = surgery  4 = other therapy  5 = Stop Protocol | **Patient outcome**  1 = no sequelae  2 = sequelae   1. = death 2. = ongoing | **Date of Resolution**  (dd/mmm/yyyy) |
| --- | --- | --- | --- | --- | --- | --- |
|  |  |  |  |  |  |  |
|  |  |  |  |  |  |  |
|  |  |  |  |  |  |  |
|  |  |  |  |  |  |  |
|  |  |  |  |  |  |  |
|  |  |  |  |  |  |  |
|  |  |  |  |  |  |  |
|  |  |  |  |  |  |  |

**FORM 7: SIGN OFF SHEET:**

# Sign Off Sheet

**This sheet must be completed. By signing this page the parties state that the forms have been reviewed and are deemed complete and accurate**.

**FORM 8: SIGN OFF SHEET**

Case Report Form to be signed off when the data has been checked as accurate and complete.

**Research Assistant:** ________________________________**Date:**_________________________

**Site Investigator:** ________________________________**Date:**_________________________

**PRISM III**
