# Supplementary material for: Music Use for Sedation in Critically ill Children (MUSiCC trial): study protocol for a pilot randomized controlled trial
Source: Pilot Feasibility Stud. 2020 Feb 25;6:31. doi: 10.1186/s40814-020-0563-x (PMC7043021; doi:10.1186/s40814-020-0563-x)
Supplement: Supplementary file 3 — Additional file 3. Informed consent. [file 40814_2020_563_MOESM3_ESM.doc]

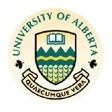
**DEPARTMENT OF PEDIATRICS**

**FACULTY OF MEDICINE AND DENTISTRY**

Title of Research Study **MUSiCC: Pilot randomized controlled trial on Music Use for Sedation In Critically ill Children.**

Principal Investigator(s): Gonzalo García Guerra MD MSc

Co-Investigator(s): Ari Joffe, Allan de Caen MD, Lisa Hartling PhD, Hsing Jou MD, Sunita Vohra MD.

Background: Your child has been admitted to the intensive care unit (PICU) because of the severity of his/her illness and/or to recover after surgery. Very sick (“critically ill”) children admitted to the PICU commonly suffer from discomfort, pain and stress, related to their illness or to the interventions that will help them to recover (catheters, chest tubes, and breathing tubes for mechanical ventilation). Management of pain and stress is extremely important in PICU as it provides comfort and prevents children from accidentally removing equipment that is necessary for their recovery. Pain and agitation are usually treated with narcotics and/or sedatives. However, these drugs can have significant side effects, including low blood pressure, weakness, confusion, breathing problems, and withdrawal symptoms upon discontinuation. Interventions that don’t involve medications, decreasing noise/lights and listening to music, can provide comfort without the side effects of narcotics and sedatives. Music has been successfully used as a sedative in children undergoing surgical procedures and in critically ill adults. We have also found that noise in the PICU is associated with discomfort and the need for extra doses of sedation. Whether music and noise cancellation can also provide comfort to critically ill children is unknown. We want to find out if music can provide comfort and reduce (not avoid) the use of sedatives and narcotics, and reduce these medication’s side effects. In our proposed study, children will be assigned to one of three groups: standard care (no music), music provided with headphones, and headphones with no music (noise cancellation). The music provided will be chosen by a pediatric music therapist. The music or noise cancellation will be provided 3 times a day. The amount of sedatives and narcotics used in each group will be recorded and compared. The need for these medications will be up to the doctors and nurses treating these children and will not depend on the study.

Purpose: We are asking you to allow your child to participate in a research study to find out if music and noise cancellation can provide comfort, help to reduce the need for sedatives and narcotics, and reduce medication side effects in critically ill children.

Procedures: Participating in this study will involve:

1. Your child will be randomized to the one of the “intervention groups” or the “control group” of the study. Randomization is like flipping a coin; this means that your child has an equal chance of being in one of the “intervention groups” or the “control group”. One of the intervention groups will receive *music* with the use of headphones, while the second intervention group will receive *noise cancellation* headphones but without music; the “control group” will not receive headphones (*standard of care*). Neither you nor your physician can choose or know which group your child is randomized to. This "blinding" is necessary to fairly test the intervention. In an emergency, your physician can immediately find out which of the study groups your child has been assigned to.
2. 24-48 hours after admission to the PICU your child will be started on the assigned intervention (music/noise cancellation/control).
3. In the *music* and *noise cancellation* groups the intervention will be delivered during 30 minutes three times a day.. The control group will receive usual care. Children will be assessed with the Sedation Behavior Scale (SBS) before and during the intervention. Signs of agitation or an increase in the SBS will indicate failure of the intervention. Patients will remain on protocol for a maximum of 7 days as long as they are on invasive mechanical ventilation. A parent survey will be conducted at the end of the study.
4. *Music* will be delivered with the use of noise cancellation headphones and an iPod Touch. Music selection will consist on classic music chosen by a music therapist based on the patient’s age. The music volume will be limited to 45-65 dB. The headphones used in the study also have a maximum volume limit of 85dB to provide extra safety.
5. N*oise cancellation* will be provided with the same headphones connected to an iPod with a silent recording.
6. The iPods provided will contain music (music group) and a sham music list (noise cancellation group) so you and the medical team do not know which group your child is in. This “blinding” is done to make sure that the results of the study are not being affected by knowing who gets the intervention. Only the research nurse will know which group your baby is in.
7. If your child appears upset or uncomfortable during the intervention (music or noise cancellation) the bedside nurse will remove the headphones.
8. The study will last 7 days or until your child is discharged from the PICU. During this time we will record some clinical information from your child’s hospital chart. This information is on the hospital chart, and will not need any extra interventions or blood work. This study will not affect any of the care your child receives in the PICU.
9. At the end of the study we will provide you with a short survey (5 minutes) so we can obtain your opinion about the intervention.

Possible Benefits: There may not be any direct benefit to your child for taking part in this study. New information about the use of music and noise cancellation in PICU will be obtained from your child’s participation in this study. This new information may benefit other children in the future.

Possible Risks: Your child may have some discomfort during the intervention, and if so, the intervention will be discontinued by the bedside nurse. Regardless of which group your child is assigned to, he/she will receive the standard of care for his/her illness and we do not expect any side-effects due to the study. This study will not affect any of the treatments or care given by the doctors and nurses to your child during the PICU stay.

Confidentiality: Personal health records relating to this study will be kept confidential. Any research data collected about your child during this study will identify your child only by his/her initials and a coded number. Your child’s name will not be disclosed outside the research clinic. Any report published as a result of this study will not identify your child’s name.

For this study, the study doctor may need to access your child’s personal health records for health information such as past medical history and test results. He/she may also need to contact your child’s pediatrician and your child’s other health care providers to obtain additional medical information. The health information collected as part of this study will be kept confidential unless release is required by law, and will be used only for the purpose of the research study. By signing the consent form you give permission to the study staff to access any personally identifiable health information which is under the custody of other health care professionals as deemed necessary for the conduct of the research. In addition to the investigators(s), people from the Health Research Ethics Board or University of Alberta may have access to your child’s personal health records to monitor the research and verify the accuracy of study data.

By signing the consent form you give permission for the collection, use and disclosure of your child’s medical records. At the University of Alberta, study information is required to be kept for 5 years. Even if you withdraw your child from the study, the medical information which is obtained from your child for study purposes will not be destroyed. You have a right to check your child’s health records and request changes if your child’s personal information is incorrect.

Voluntary Participation: If you agree to participate, you are free to withdraw your child from the research study at any time, and your child’s continuing medical care will not be affected in any way. If the study is not undertaken or if it is discontinued at any time, the quality of your child’s medical care will not be affected. If any knowledge gained from this or any other study becomes available which could influence your decision to continue your child in the study, you will be promptly informed.

Compensation for Injury: If your child becomes ill or injured as a result of being in this study, he/she will receive necessary medical treatment, at no additional cost to you. By signing this consent form you are not releasing the investigator(s), institution(s) and/or sponsor(s) from their legal and professional responsibilities.

Contact Names and Telephone Numbers:

If you have concerns about your or your child’s rights as a study participant, you may contact the University of Alberta Research Ethics Office, at 780 492-2615. This office has no affiliation with the study investigators.

Please contact any of the individuals identified below if you have any questions or concerns:

**DEPARTMENT OF PEDIATRICS**


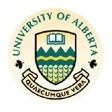
**FACULTY OF MEDICINE AND DENTISTRY**

Title of Research Study **MUSiCC: Pilot randomized controlled trial on Music Use for Sedation In Critically ill Children.**

Principal Investigator(s): Gonzalo García Guerra MD, 780-407-1673, 780-445-5963 (pager)

Co-Investigator(s): Ari Joffe, Allan de Caen MD, Lisa Hartling PhD, Hsing Jou MD, Sunita Vohra MD.

|  | | Yes | No |
| --- | --- | --- | --- |
| Do you understand that you have been asked to have your child in a research study? | | 🞏 | 🞏 |
| Have you read and received a copy of the attached Information Sheet? | | 🞏 | 🞏 |
| Do you understand the benefits and risks involved in your child taking part in this research study? | | 🞏 | 🞏 |
| Have you had an opportunity to ask questions and discuss this study? | | 🞏 | 🞏 |
| Do you understand that you are free to withdraw from the study at any time, without having to give a reason and leaving the study will not affect your child’s future medical care? | | 🞏 | 🞏 |
| Has the issue of confidentiality been explained to you? | | 🞏 | 🞏 |
| Do you understand who will have access to your child records, including personally identifiable health information? | | 🞏 | 🞏 |
| Do you wish to be contacted at the end of the study so we can provide you with information regarding the study results as well as letting you know which intervention group your child was randomized to?  If so, please provide your email or postal address: ________________  _____________________________________________________________ | | 🞏 | 🞏 |
| Who explained this study to you? |  | |  |

I agree for _____________________________ (child’s name) to take part in this study.

Parent/Guardian name Parent/Guardian signature Date

*I believe that the person signing this form understands what is involved in the study and voluntarily agrees to participate.*

Signature of the person who obtained consent Date

**THE INFORMATION SHEET MUST BE ATTACHED TO THIS CONSENT FORM AND A COPY GIVEN TO THE RESEARCH PARTICIPANT**
